# Physical Activity, Sedentary Behavior, Diet, and Habits in Older Patients With Colorectal Cancer

**DOI:** 10.7759/cureus.96382

**Published:** 2025-11-08

**Authors:** Alexis Aguilar Blancas, María T Alvarez Bañuelos, María del Pilar Ramírez Díaz, Rebeca Garcia Román, Clara L Sampieri

**Affiliations:** 1 Teaching and Research, Centro de Alta Especialidad, "Dr. Rafael Lucio", Xalapa, MEX; 2 Public Health Institute, Universidad Veracruzana, Xalapa, MEX; 3 Nutrition, Universidad del Istmo, Oaxaca, MEX

**Keywords:** colorectal cancer, dietary patterns, occupation, physical activity, sedentary behaviour

## Abstract

Background: Colorectal cancer (CRC) is characterized by high incidence and mortality rates among men and women in Mexico and worldwide. Its multifactorial etiology is strongly associated with environmental factors, particularly among older adults, including physical activity, sedentary behavior, and diet.

Methodology: We conducted an analytical cross-sectional study using a food frequency questionnaire validated for the Mexican population and a tool developed by the World Health Organization (WHO) to assess risk factors for chronic diseases and physical activity levels.

Results: The overall prevalence of a sedentary lifestyle was 55.6%, although most participants reported moderate levels of physical activity. Three dietary patterns were identified: *healthy*, *carbohydrates and protein*, and *fats and sugars*. Women exhibited greater adherence to the healthy dietary pattern. Multivariate analysis indicated that farm workers were more likely to achieve adequate physical activity levels compared to office workers (odds ratio (OR) = 0.080, 95% confidence interval (CI) 0.014-0.461). Younger participants (under 50 years) (OR = 0.239, 95% CI 0.059-0.966) and those diagnosed with colon cancer (OR = 0.237, 95% CI 0.066-0.848) also had higher activity levels compared to patients with rectal cancer.

Conclusions: Physical activity levels were influenced by occupation, cancer type, and age, underscoring the importance of tailored interventions. Two of the three dietary patterns were classified as healthy, with one providing greater dietary diversity and balance. These findings emphasize the need to promote healthier lifestyles among patients with CRC to improve clinical outcomes and reduce sedentary behavior.

## Introduction

Colorectal cancer (CRC) is one of the most frequently diagnosed types of cancer among males and females. According to the Pan American Health Organization (PAHO), it is estimated that by 2030, the incidence of CRC will increase by 70% in men and 80% in women in the American continent [1,2]. In Mexico, a study by Espinosa-Tamez et al. highlighted that CRC is a significant public health concern, ranking among the leading causes of death from malignant neoplasms [3].

The etiology of CRC is known to be multifactorial, with environmental risk factors such as diet, physical activity, sedentary behavior, and genetic factors playing a key role in its development [4,5]. Regarding diet, various investigations have shown that unhealthy patterns, characterized by a high intake of animal-source foods, processed meats, and saturated fats, labeled as the Western dietary pattern, are associated with a higher risk of developing CRC. Conversely, healthy patterns, characterized by a proper intake of fiber, fruits, vegetables, and whole grains, have demonstrated protective effects [6-8].

Sedentary behavior has also been linked to CRC risk, with evidence suggesting an association with colorectal adenoma recurrence. Conversely, adequate levels of physical activity appear to confer protective benefits [9,10].

Despite the growing impact of CRC in Mexico over the last decade, there is still a lack of comprehensive scientific evidence addressing dietary patterns, physical activity levels, and sedentary behavior at both state and regional levels. In this context, the present study aims to explore these aspects among patients with CRC, considering the broader framework of the Mexican healthcare system.

## Materials and methods

Study design and setting

A descriptive and analytical cross-sectional study was conducted at a tertiary care oncology center in Xalapa, Veracruz, Mexico. The institution provides specialized care to patients referred from primary and secondary healthcare facilities, as well as to individuals without social security coverage. Its broad regional reach and access to updated clinical records made it a suitable setting for epidemiological research involving colorectal cancer patients.

The study population included adult patients with histologically confirmed diagnoses of colon or rectal cancer who attended outpatient consultations at the “Dr. Miguel Dorantes Mesa” State Cancer Center between August 2022 and January 2023. Patients with metastatic disease, limited mobility, or those hospitalized during the study period were excluded.

Sample size

The sample size was estimated using the annual average of colorectal cancer cases treated at the State Cancer Center (2020-2021), based on institutional data (Revista Médica de la Universidad Veracruzana, 2021)[11]. Assuming an expected prevalence of 8.1%, a 7% margin of error, and a 95% confidence level, the minimum required sample was 59 patients. The 7% margin of error reflects the accepted level of statistical precision for estimating the population proportion of patients with CRC treated at the hospital, which is appropriate for exploratory descriptive studies focused on prevalence rather than inferential group comparisons.

A total of 72 participants were included, enhancing the statistical power of the analyses. Participants were selected through consecutive non-probabilistic sampling during routine oncology consultations.

Data collection instrument

Sociodemographic and clinical data were collected using the WHO STEPwise approach to NCD risk factor surveillance (STEPS) instrument [12], which includes modules on physical activity and sedentary behavior. Physical activity was assessed using the International Physical Activity Questionnaire (IPAQ, long version), which has demonstrated good convergent validity and reliability in Spanish-speaking populations, including Mexico, with Cronbach’s alpha values of 0.80 and test-retest correlation coefficients of 0.83. Dietary intake was evaluated using the Food Frequency Questionnaire (FFQ) from the 2018 National Health and Nutrition Survey (ENSANUT), validated in Mexican adolescent and adult populations [13-14]. This instrument showed an average intraclass correlation coefficient (ICC) of 0.54 for reproducibility and energy-adjusted correlation coefficients ranging from 0.40 to 0.60 for validity compared to repeated 24-hour recalls. To enhance portion size estimation and minimize recall bias, a standardized food model kit was employed.

Measuring physical activity

Physical activity was classified according to the criteria and cutoffs established by international guidelines from the World Health Organization (WHO) and the American College of Sports Medicine (ACSM). Total MET-minutes per week are categorized into three levels: high (>3,000 MET-minutes/week, or > 1,500 MET-minutes/week with at least three days of vigorous activity), moderate (>600 MET-minutes per week), and low <600 MET-minutes/week).

In this study, we consistently used the terms physically active and physically inactive based on IPAQ classification: individuals accumulating <600 MET-minutes/week were considered physically inactive, while those meeting or exceeding this threshold were classified as physically active [13,15].

Sedentary behavior was defined as engaging in activities requiring ≤1.5 METs in a sitting, reclining, or lying position. Participants reporting >5 hours/day in such activities were classified as sedentary [16,17].

Dietary assessment

A 163-item Food Frequency Questionnaire, covering 15 food groups, was administered to assess participants’ dietary patterns using the previous week as the reference period. Frequency and quantity of food consumption per day and week were recorded. Total energy intake and net grams were then calculated, and foods were grouped according to nutritional similarity.

Dietary patterns were identified using factor analysis and principal component analysis (PCA) with Varimax rotation. Factor loadings were calculated for 14 food groups, yielding a Kaiser-Meyer-Olkin (KMO) value of 0.581 and a statistically significant Bartlett’s test (*P* = 0.001). Based on eigenvalues >1.3, three factors were extracted, explaining 41.9% of the total variance: Factor 1 (16.12%), Factor 2 (14.18%), and Factor 3 (11.57%).

Variables

Sociodemographic variables included schooling (no education, primary, secondary, and higher education), employment status (employed vs. unemployed), and place of residence, defined as rural (<15,000 inhabitants) or urban (≥15,000 inhabitants) [18], according to the classification established by INEGI (National Institute of Statistics and Geography). Socioeconomic status was defined according to CONEVAL (National Council for the Assessment of Social Development Policy) [19].

Clinical variables included excess weight (BMI ≥25 kg/m²), according to WHO criteria; alcohol consumption (≥1 standard drink, ≈14 g/day); smoking status (current, former, or non-smoker, with smoking defined as ≥1 cigarette per day); degree of consanguinity (first, second, or third degree); and the presence of type 2 diabetes mellitus or hypertension, determined through review of medical records, clinical history, physician diagnoses, and laboratory results.

Statistical analysis

Data were analyzed using Epidat (version 4.2, July 2016) and IBM SPSS Statistics for Mac (version 23.0, IBM Corp., Armonk, NY). Descriptive statistics summarized population characteristics. Continuous variables were expressed as means ± standard deviations (SDs) after normality testing, and categorical variables as percentages, compared using chi-square or Fisher’s exact tests. Univariate analysis examined associations between physical activity level (active vs. inactive) and sociodemographic or clinical variables. Crude odds ratios (ORs) and 95% confidence intervals (CIs) were calculated.

A multivariate logistic regression model was then applied to identify factors independently associated with adequate physical activity, defined as >600 MET-minutes/week according to WHO criteria. Independent variables included age (<50 vs. ≥50 years), occupation (agricultural vs. non-agricultural), tumor location (colon vs. rectal cancer), comorbidities (type 2 diabetes mellitus and hypertension), and adherence to dietary patterns. Both unadjusted and adjusted ORs with 95% CIs were reported.

Finally, Multiple Correspondence Analysis (MCA) was performed to visually explore associations among categorical variables, including dietary patterns, sex, physical activity level, sedentary behavior, and comorbidities. This multivariate technique enables the graphical representation of relationships among non-continuous variables, providing an overview of underlying association patterns.

Ethical considerations

The study was approved by the Research Ethics Board of Hospital Regional de Xalapa “Dr. Luis F. Nachón” and authorized by the “Dr. Miguel Dorantes Mesa” State Cancer Center (reference number C.E.I./2022/01). All participants provided written informed consent before enrollment.

## Results

Baseline characteristics

Of 77 patients who met the inclusion criteria to participate, 3 declined, and 2 were seriously hospitalized. A total of 72 participants were recruited. Among them, 41 (56.9%) were male and 31 (43.1%) were female, with ages ranging from 29 to 85 years. Most participants (63, 87.5%) were employed, while 9 (12.5%) were unemployed. Regarding education, 51 (70.9%) had basic or no education, whereas 21 (29.2%) had completed high school (Table 1).

**Table 1:** Sociodemographic characteristics and comorbidities for patients with colorectal cancer. SD, standard deviation

Variable		General (*n* = 72)	Sex	
			Male (*n *= 41)	Female (*n* = 31)
Age (years) (mean ± SD)		53.81 ± 13.07	52.17 ± 12.73	54.65 ± 13.67
Diagnostic, *n* (%)				
Colon		48 (66.7)	22 (53.7)	26 (83.9)
Rectal		24 (33.3)	19 (46.3)	5 (16.1)
Diagnostic time, *n* (%)				
<1 year		15 (20.8)	9 (22.0)	6 (19.4)
>1 year		57 (79.2)	32 (78.0)	25 (80.6)
Place of residence, *n* (%)				
Rural		39 (54.2)	26 (63.4)	13 (41.9)
Urban		33 (45.8)	15 (36.6)	18 (58.1)
Monthly income, *n* (%)				
Less than $5,000		54 (75)	26 (63.4)	28 (90.3)
$5,001 to $10,000		16 (22.2)	14 (34.1)	2 (6.5)
More than $10,000		2 (2.8)	1 (2.4)	1 (3.2)
Occupation, *n* (%)				
Housewife		19 (26.4)	0 (0)	19 (61.3)
Farmer		19 (26.4)	18 (43.9)	1 (3.2)
Other		12 (16.7)	11 (26.8)	1 (3.2)
Unemployed		9 (12.5)	4 (9.8)	5 (16.1)
Office worker		7 (9.7)	5 (12.2)	2 (6.5)
Trader		6 (8.3)	3 (7.3)	3 (9.7)
Schooling, *n* (%)				
No education		13 (18.0)	9 (22)	4 (12.9)
Basic education		38 (52.8)	20 (48.8)	18 (58.1)
Secondary education		12 (16.7)	6 (14.6)	6 (19.4)
Higher education		9 (12.5)	6 (14.6)	3 (9.7)

A substantial proportion of participants reported a family history of cancer, most commonly among first-degree relatives. The predominant cancer types were colorectal, breast, and cervical cancer (Table 1). Adherence rates were 41.7% for Pattern 1, 29.9% for Pattern 2, and 29.9% for Pattern 3 (Table 1). Women showed greater adherence to Pattern 1, while men were more frequently associated with Pattern 2 (Appendix).

The most frequent comorbidities in the study population included type 2 diabetes mellitus, hypertension, and obesity. In addition, histories of alcohol consumption and smoking were recorded, showing statistically significant differences between male and female participants (*P* < 0.05).

Dietary patterns

Three distinct dietary patterns were extracted through factor and PCAs, guided by the eigenvalue criterion (>1.3) and interpretation of the scree plot. Together, these patterns explained 39.9% of the total variance. Pattern 1 (eigenvalue = 2.46; 17.6%) reflected frequent intake of legumes, cereals, tubers, fruits, vegetables, and chicken. Pattern 2 (eigenvalue = 1.65; 11.8%) was associated with consumption of maize-based products, fish, and sugar-free beverages. Pattern 3 (eigenvalue = 1.47; 10.5%) encompassed dietary habits characterized by animal fats, sugary drinks, and processed or high-calorie foods (Table 2).

**Table 2:** Rotated component matrix for patients with colorectal cancer. Data extraction technique: Principal components analysis. Rotation method: Varimax with Kaiser normalization. Factor loading values >0.30 were considered to represent a meaningful strength of association. Bartlett’s χ² = 116.65 (*P* < 0.0001), degrees of freedom (df) = 66.

Food groups	Components		
	Pattern 1	Pattern 2	Pattern 3
Legumes	0.743	-	-
Cereals and tubers	0.736	-	-
Fast food	-	-	-
Maize dishes	-	0.541	-
Tortilla	-		0.350
Fish	-	0.617	-
Sugar-free beverages	-	0.583	-
Fruits and vegetables	0.618	-	-
Pasta	-	-	-
Chicken	0.507	-	-
Animal fats	-	-	0.598
Sugary beverages	-	-	0.600

Multicollinearity was assessed before conducting the PCA. The determinant of the correlation matrix was greater than 0.00001, indicating no presence of excessive multicollinearity. Additionally, sampling adequacy was confirmed with a Kaiser-Meyer-Olkin (KMO) value of 0.581 and a significant Bartlett’s test of sphericity (χ² = 116.65, *P* < 0.0001). These indicators confirmed the suitability of the data for factor analysis. The corresponding information has been included in the Methods section (Table 3).

**Table 3:** Comparison of dietary patterns with sociodemographic and clinical characteristics of patients with colorectal cancer. BMI: Low, <18.5 kg/m^2^; normal, 18.5 to 24.9 kg/m^2^; overweight, 25 to 29.9 kg/m^2^; obesity, >30 kg/m^2^.

Variables	Dietary pattern		
	Healthy	Carbohydrates and proteins	Fats and sugars
	*n* (%)	*n* (%)	*n* (%)
Sex			
Male	15 (36.6)	8 (19.5)	18 (43.9)
Female	15 (48.4)	13 (41.9)	3 (9.7)
Age			
Under 50 years	12 (41.4)	8 (27.6)	9 (31)
50 years or older	18 (41.9)	13 (30.2)	12 (27.9)
Place of residence			
Rural	19 (48.7%)	9 (23.1)	11 (28.2)
Urban	11 (33.3)	12 (36.4)	10 (30.3)
Occupation			
Employed	25 (39.7)	20 (31.7)	18 (28.6)
unemployed	5 (55.6)	1 (11.1)	3 (33.3)
Monthly income			
Less than $5,000	27 (50)	13 (24.1)	14 (25.9)
More than $5,000	3 (16.7)	8 (44.4)	7 (38.9)
Level of schooling			
Basic education	21 (41.2)	12 (23.5)	18 (35.3)
Secondary education	9 (42.9)	9 (42.9)	3 (14.3)
Patient diagnosis			
Colon	22 (45.8)	15 (31.3)	11 (22.9)
Rectal	8 (33.3)	6 (25)	10 (41.7)
Diagnostic time			
<1 year	8 (53.3)	3 (20)	4 (26.7)
>1 year	22 (38.6)	18 (31.6)	17 (29.8)
Body mass index (BMI)			
Low + normal	13 (48.1	8 (29.6)	6 (22.2)
Overweight + Obesity	17 (37.8)	13 (28.9)	15 (33.3)
Type 2 diabetes mellitus			
Yes	8 (53.3)	3 (20)	4 (26.7)
No	22 (38.6)	18 (31.6)	17 (29.8)
Hypertension			
Yes	8 (50)	4 (25)	4 (25)
No	22 (39.3)	17 (30.4)	17 (30.4)

**Figure 1:**
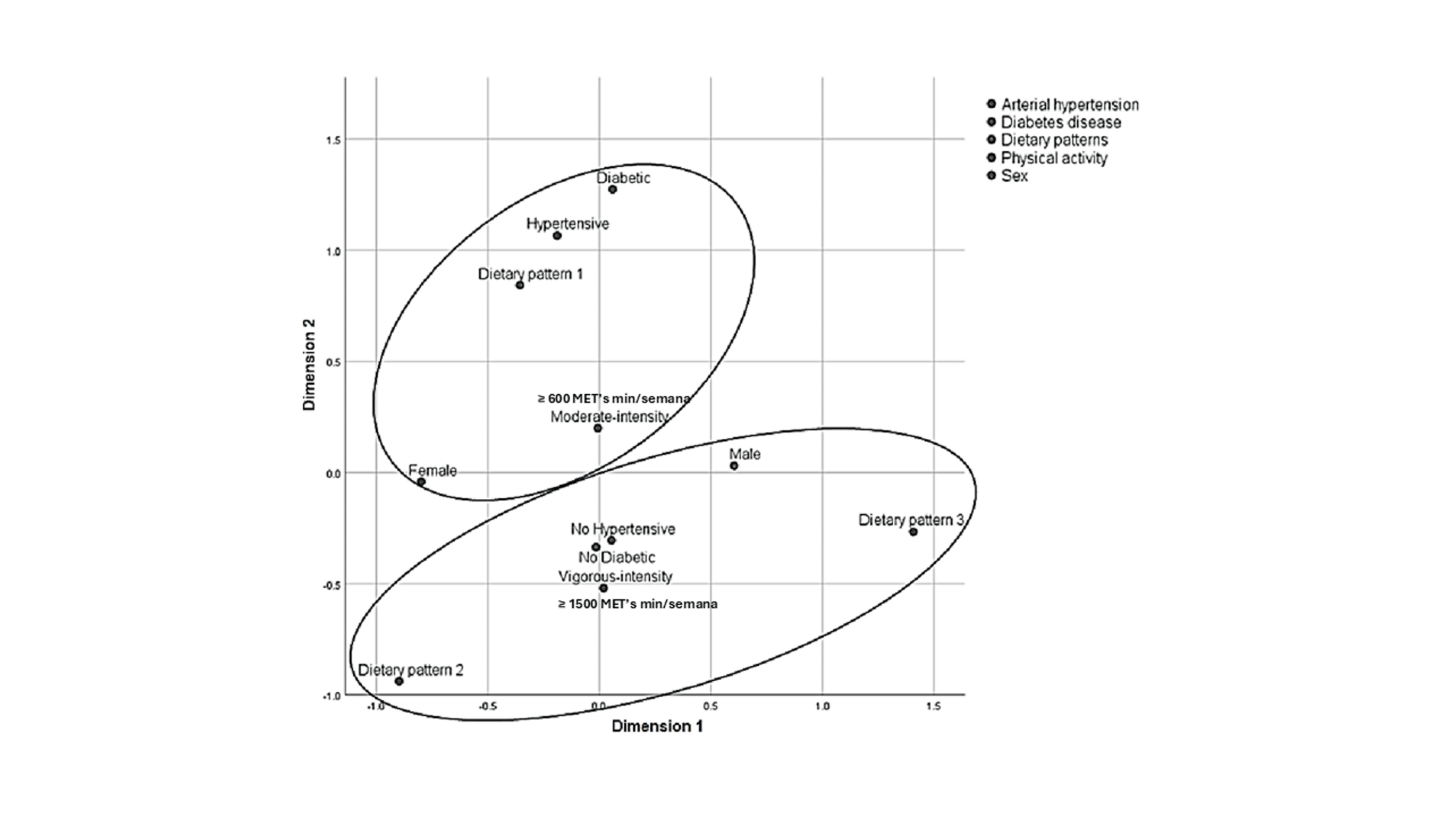
Multiple correspondence analysis of dietary patterns by sex, physical activity level, type 2 diabetes, and hypertension in older patients with colorectal cancer.

Correspondence analysis suggested independence among variables; however, graphical exploration revealed trends. Male participants tended to engage in vigorous physical activity, had fewer comorbidities, and adhered more to Patterns 2 and 3. In contrast, female participants displayed moderate activity levels, greater adherence to Pattern 1, and a higher prevalence of type 2 diabetes mellitus and hypertension (Figure 1).

Physical activity

Univariate Analysis of Physical Activity Levels

Overall, 20 (27.8%) participants reported engaging in vigorous physical activity (>1,500 MET-minutes/week), 30 (41.7%) in moderate activity (>600 MET-minutes/week), and 22 (30.6%) in low activity (<600 MET-minutes/week). The overall prevalence of a sedentary lifestyle was 40 (55.6%). Nonetheless, a considerable proportion also reported moderate physical activity, particularly among men (24, 58.5%) and women (16, 51.6%).

Participants younger than 50 years and those currently employed were significantly more likely to be physically active (OR = 4.09; 95% CI 1.29-13.0; *P* = 0.018 and OR = 6.40; 95% CI 1.43-28.6; *P* = 0.008, respectively). In addition, individuals with colon tumors showed a trend toward higher activity levels (OR = 2.91; 95% CI 0.99-8.54; *P* = 0.010). Conversely, the presence of type 2 diabetes mellitus and hypertension was associated with lower physical activity (OR = 3.87; 95% CI 1.22-12.3; *P* = 0.021 and OR = 3.31; 95% CI 1.00-10.9; *P* = 0.038, respectively). 

No significant associations were found with sex, rural residence, educational level, time since diagnosis, or adherence to a healthy dietary pattern. Notably, 40 (55.6%) participants reported a decrease in physical activity during the COVID-19 pandemic (Table 4).

**Table 4:** Analysis of physical activity levels with sociodemographic and clinical factors in patients with colorectal cancer. Distribution of sociodemographic and clinical variables according to physical activity level (active vs. inactive). Frequency and percentage are shown for each category. Odds ratios (OR), 95% confidence intervals (95% CIs). An OR > 1 indicates a higher likelihood of physical activity in the exposed group. Statistical significance was defined as *P* < 0.05.

Variables	Physical activity level		OR	95% CI	*P*-value*
	Active (*n* = 51), *n* (%)	Inactive (*n* = 21), *n* (%)			
Sex: Male	29 (56.8)	12 (57.1)	1.02	0.37-2.81	0.980
Age < 50 years	25 (49.0)	4 (19.0)	4.09	1.29-13.0	0.018
Employed	48 (94.1)	15 (71.4)	6.40	1.43-28.6	0.008
Rural	29 (56.8)	10 (47.6)	0.69	0.26-1.85	0.474
Basic education	34 (66.7)	17 (81.0)	2.17	0.63-7.45	0.225
Tumor colon	37 (72.5)	10 (47.6)	2.91	0.99-8.54	0.01
Diagnostic < 1 year	10 (19.6)	5 (23.8)	0.80	0.22-2.89	0.750
Type 2 diabetes mellitus	7 (13.7)	8 (38.1)	3.87	0.22-2.89	0.021
Hypertension	8 (15.7)	8 (38.1)	3.31	1.00- 10.9	0.038
Healthy pattern	38 (74.5)	13 (61.9)	1.84	0.61- 5.56	0.285

Multivariate analysis revealed that participants engaged in physically demanding occupations were significantly more likely to meet the recommended physical activity levels compared with those in administrative or office-based roles (OR = 13.03; 95% CI 2.22-76.42; *P* = 0.004). Moreover, patients with colon cancer had higher odds of achieving adequate physical activity levels than those diagnosed with rectal cancer (OR = 4.29; 95% CI 1.15-15.95; *P* = 0.030). Conversely, hypertension and dietary patterns were not significantly associated with physical activity after adjusting for potential confounders, including overweight/obesity and time since diagnosis (Table 5).

**Table 5:** Multivariate logistic regression analysis of physically active levels in relation to sociodemographic and clinical variables among patients with colorectal cancer. Dependent variable: recommended level of MET minutes per week. *P*-value ≤ 0.05 was considered statistically significant. The logistic regression model was adjusted using the *forward conditional method*. OR, odds ratio; CI, confidence interval

Covariates	Unadjusted model (OR (95% CI))	P	Adjusted model (OR (95% CI))	P
Model				
Age <50 years	0.245 (0.072-0.829)	0.018	3.12 (0.64-15.29)	0.161
Working in agriculture	6.4 (1.425-28.7)	0.015	13.03 (2.22-76.42)	0.004
Tumor localization (colon)	0.416 (0.145-1.195)	0.099	4.29 (1.15-15.95)	0.030
Type 2 diabetes mellitus	0.259 (0.079-0.848)	0.029	-	-
Hypertension	0.302 (0.095-0.964)	0.038	-	-
Dietary patterns	1.799 (0.609-5.31)	0.393	-	-

Living in rural areas and frequent fruit consumption were also associated with higher physical activity levels after adjustment for confounders.

## Discussion

One of the highlights of this study was the assessment of post-diagnostic sedentary behavior, which showed a high prevalence (5, 55.6%). Although there is no standard definition of sedentary behavior as sitting for more than five hours per day, several studies suggest that spending more than four hours daily in sedentary activities can have significant adverse health effects [16,17,20]. The remaining patients reported moderate activity levels, particularly those with physically demanding occupations such as agriculture. However, physical activity represents only one component of a broader set of protective factors. In our analysis, moderate-to-high levels of physical activity were associated with a lower risk of a sedentary lifestyle, particularly with respect to age (*P* < 0.005).

Because data collection took place during the COVID-19 pandemic, we included an open-ended question (not shown in the results). Patients reported decreased activity levels due to pandemic-related restrictions. These findings are consistent with Byrne et al., who reported that one-third of patients with cancer (32%) reduced their regular exercise, while only 11% increased it. Similarly, Andrilla et al. found that reduced activity was more common among unemployed, retired, or actively treated patients [21,22].

Another factor to consider is that most participants were from rural areas, engaged in agricultural work, had basic education, and were presented with advanced-stage cancer. Consistent with Kindred et al. and Andrilla et al., our results underscore the link between rurality, persistent poverty, low levels of education, and late-stage cancer diagnosis. Longitudinal studies have shown that lower sedentary behavior and higher levels of moderate to vigorous activity benefit CRC survivors. Clinical trials also emphasize identifying survivors with high sedentary behavior to reduce prolonged inactivity and promote moderate physical activity. Suggests that insufficient exercise after diagnosis increases the risk of CRC relapses and all-cause mortality [21,23-24].

Since we identified three dietary patterns, the variation in dietary habits among CRC survivors became evident. Our findings suggest associations, but not causality, due to the cross-sectional design. However, evidence from Chen et al. and Zhu et al. supports the benefits of plant-based diets and reduced consumption of animal products. Such diets, rich in phytochemicals with antioxidants and anti-inflammatory properties, may improve prognosis by reducing CRC incidence and enhancing intestinal transit [7,25].

Interestingly, the dietary patterns observed here were less diverse and limited to certain food groups, reflecting the socioeconomic and rural context of our population. This lack of diversity may also be explained by the absence of nutritional counseling and the timing of data collection during the pandemic. Rodriguez-Ramirez et al. and Gaona-Pineda et al. documented similar pandemic-related dietary changes in Mexican households: 66% reporting reduced meat/fish consumption and over 50% reporting decreased intake of fruits and vegetables [26,27].

Sex differences were evident in dietary habits: females adhered more to fruit- and vegetable-rich patterns, while males followed diets richer in animal fats and sugary beverages. These findings underscore the importance of considering gender differences when designing nutritional strategies for CRC survivors. For example, Pou et al. recommended moderating egg intake in men and the consumption of mayonnaise, oils, and pastries in women [28]. These results are consistent with previous studies showing that males consume more sugary drinks than females. Designing nutrition and health initiatives should consider gender stereotypes and specific unmet needs, as reflected in the differing perceptions between males and females. Our findings also confirm previous reports of higher sugary consumption in males. In contrast, Zhu et al. found men to follow “prudent” patterns, underscoring that sex-specific dietary behaviors may vary by population and environmental context [25,29].

Our sample was relatively homogeneous, with most participants coming from low socioeconomic backgrounds, an average age of 54 years, and a predominantly rural origin. Under these conditions, no significant differences in dietary patterns or sedentary behavior were found across subgroups. Thus, multiple correspondence statistical analysis proved useful in exploring complex categorical variables, such as physical level and comorbidities. These results may inform targeted public health strategies and nutrition programs that consider sex-specific needs. In particular, the trend toward higher sugar intake among men warrants tailored interventions.

Strengths and limitations 

A major strength of this study lies in its focus on dietary patterns and physical activity among patients with CRC in the Mexican context, where empirical evidence remains limited. Considering Mexico’s cultural and gastronomic diversity, documenting these behavioral patterns among CRC survivors provides valuable and novel insights.

However, several limitations should be acknowledged. The cross-sectional design precludes causal inference. Recall bias in dietary assessment was inevitable but minimized by focusing on frequency rather than portion size and by using validated instruments alongside standardized food models to enhance accuracy. Additionally, the relatively small sample size and the use of non-probability sampling limit the generalizability of the findings.

It is also recognized that the sample size estimation was initially based on colorectal cancer prevalence, which may not be optimal for a population already diagnosed with CRC. Future research should estimate sample size based on the prevalence of exposures or outcomes under study to improve methodological precision. Moreover, subsequent studies should incorporate objective measures-such as accelerometry and dietary biomarkers strengthen the validity of physical activity and dietary assessments.

## Conclusions

This study found a high prevalence of sedentary behavior among patients with CRC in Veracruz, Mexico. Physical inactivity was associated with an increased risk of excess weight, obesity, and chronic diseases, whereas higher activity levels were linked to occupational factors and the type of cancer. Among the three dietary patterns identified, two were classified as *healthy*. Pattern 1, characterized by greater dietary diversity, was more prevalent among women, suggesting sex-related differences in beliefs and attitudes toward nutrition.

These findings highlight the importance of implementing targeted interventions to promote physical activity and healthier dietary practices among CRC survivors. Addressing sex-specific differences in nutrition and lifestyle is essential for reducing the risk of recurrence and enhancing long-term outcomes.

## Figures and Tables

**Figure 2:**
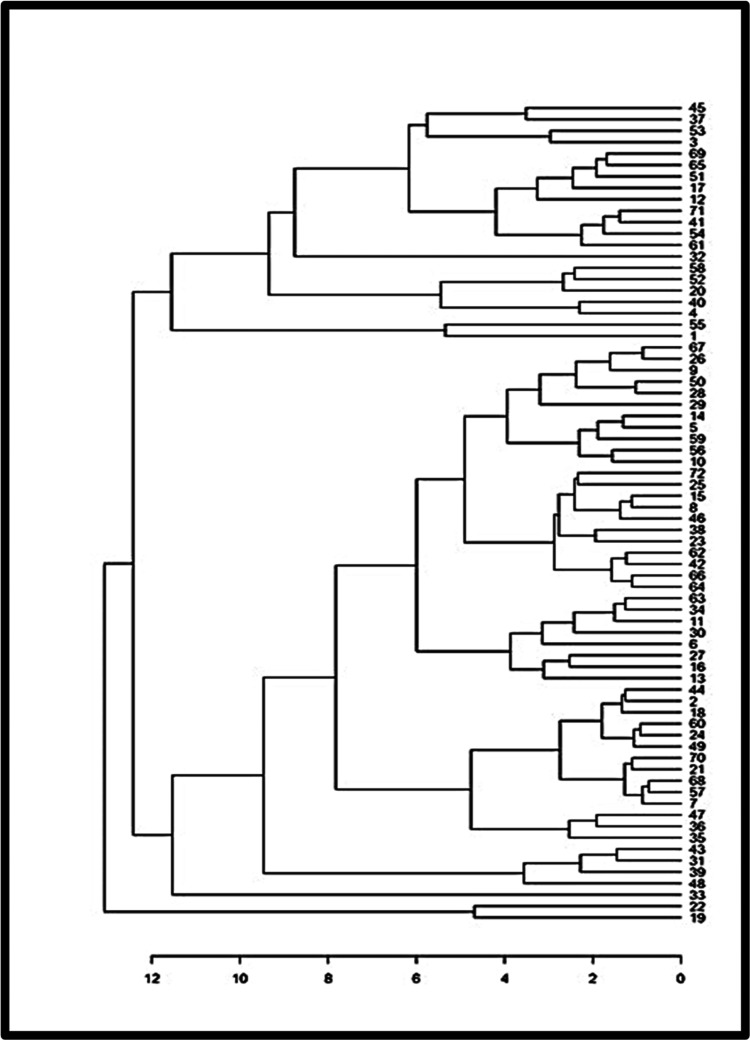
Dendrogram Three clusters for individuals.

## Appendices

Appendix 

## References

[1] Bray F, Laversanne M, Sung H, Ferlay J, Siegel RL, Soerjomataram I, Jemal A (2024). Global cancer statistics 2022: GLOBOCAN estimates of incidence and mortality worldwide for 36 cancers in 185 countries. CA Cancer J Clin.

[2] Luciani S, Nederveen L, Martinez R (2023). Noncommunicable diseases in the Americas: a review of the Pan American Health Organization's 25-year program of work. Rev Panam Salud Publica.

[3] Espinosa-Tamez P, Suazo-Zepeda E, Sánchez-Blas H (2021). National and state-level colorectal cancer mortality trends in Mexico, 1998-2018. Salud Publica Mex.

[4] Saliba W, Rennert HS, Gronich N, Gruber SB, Rennert G (2019). Red meat and processed meat intake and risk of colorectal cancer: a population-based case-control study. Eur J Cancer Prev.

[5] Thanikachalam K, Khan G (2025). Colorectal cancer and nutrition. Nutrients.

[6] Safari A, Shariff ZM, Kandiah M, Rashidkhani B, Fereidooni F (2013). Dietary patterns and risk of colorectal cancer in Tehran Province: a case-control study. BMC Public Health.

[7] Chen Z, Wang PP, Woodrow J, Zhu Y, Roebothan B, Mclaughlin JR, Parfrey PS (2015). Dietary patterns and colorectal cancer: results from a Canadian population-based study. Nutr J.

[8] Feng YL, Shu L, Zheng PF (2017). Dietary patterns and colorectal cancer risk: a meta-analysis. Eur J Cancer Prev.

[9] Molmenti CL, Hibler EA, Ashbeck EL (2014). Sedentary behavior is associated with colorectal adenoma recurrence in men. Cancer Causes Control.

[10] Oruç Z, Kaplan MA (2019). Effect of exercise on colorectal cancer prevention and treatment. World J Gastrointest Oncol.

[11] Brizio PGC, González JO: (2021). Reporte de indicadores del Centro Estatal de Cancerología de Veracruz. Revista Médica de la Universidad Veracruzana.

[12] (2024). OPS/WHO: PanAmerican STEPSwise: The PAHO / WHO Stepwise Approach to Chronic Noncommunicable Diseases Risk-Factor Surveillance. https://www.paho.org/en/documents/pahowho-step-wise-approach-noncommunicable-disease-ncd-risk-factor-surveillance-steps..

[13] Mantilla Toloza SC, Gómez-Conesa A: El Cuestionario Internacional de Actividad Física (2007). Un instrumento adecuado en el seguimiento de la actividad física poblacional. Revista Iberoamericana de Fisioterapia y Kinesiología.

[14] Denova-Gutiérrez E, Ramírez-Silva I, Rodríguez-Ramírez S, Jiménez-Aguilar A, Shamah-Levy T, Rivera-Dommarco JA (2016). Validity of a food frequency questionnaire to assess food intake in Mexican adolescent and adult population. Salud Publica Mex.

[15] Rock CL, Thomson CA, Sullivan KR (2022). American Cancer Society nutrition and physical activity guideline for cancer survivors. CA Cancer J Clin.

[16] Arem H, Pfeiffer RM, Engels EA, Alfano CM, Hollenbeck A, Park Y, Matthews CE (2015). Pre- and postdiagnosis physical activity, television viewing, and mortality among patients with colorectal cancer in the National Institutes of Health-AARP Diet and Health Study. J Clin Oncol.

[17] Morris JS, Bradbury KE, Cross AJ, Gunter MJ, Murphy N (2018). Physical activity, sedentary behaviour and colorectal cancer risk in the UK Biobank. Br J Cancer.

[18] González-Arellano S, Larralde-Corona AH, Cruz-Bello GM: (2021). El periurbano en México: Identificación y caracterización sociodemográfica y territorial.. Papeles de Población.

[19] Damián A, Damián A (2019). Pobreza y desigualdad en México: La construcción ideológica y fáctica de ciudadanías diversas y desiguales. Trimest Econ.

[20] Hatime Z, El Kinany K, Huybrechts I (2022). Association of physical activity and sedentary behavior with colorectal cancer risk in Moroccan adults: a large-scale, population-based case-control study. Asian Pac J Cancer Prev.

[21] Byrne NW, Parente DJ, Yedlinsky NT (2022). Impact of the COVID-19 pandemic on exercise habits among US primary care patients. J Am Board Fam Med.

[22] Andrilla CHA, Moore TE, Man Wong K, Evans D V (2020). Investigating the impact of geographic location on colorectal cancer stage at diagnosis: a national study of the SEER cancer registry. J Rural Health.

[23] Kindred MM, Pinto BM, Dunsiger SI (2019). Predictors of sedentary behavior among colorectal survivors. Support Care Cancer.

[24] Swain CT, Nguyen NH, Eagles T, Vallance JK, Boyle T, Lahart IM, Lynch BM (2020). Postdiagnosis sedentary behavior and health outcomes in cancer survivors: A systematic review and meta-analysis. Cancer.

[25] Zhu Y, Wu H, Wang PP (2013). Dietary patterns and colorectal cancer recurrence and survival: a cohort study. BMJ Open.

[26] Rodríguez-Ramírez S, Gaona-Pineda EB, Martínez-Tapia B, Romero-Martínez M, Mundo-Rosas V, Shamah-Levy T (2021). Inseguridad alimentaria y percepción de cambios en la alimentación en hogares mexicanos durante el confinamiento por la pandemia de Covid-19. Salud Publica Mex.

[27] Gaona-Pineda EB, Rodríguez-Ramírez S, Medina-Zacarías MC, Valenzuela-Bravo DG, Martinez-Tapia B, Arango-Angarita A (2023). Consumidores de grupos de alimentos en población mexicana. Ensanut Continua 2020-2022. Salud Publica Mex.

[28] Pou SA, Niclis C, Aballay LR (2014). Cancer and its association with dietary patterns in Córdoba (Argentina). Nutr Hosp.

[29] Laguerre E, Matthews T (2022). Association between nutrition behavior and colorectal cancer diet recommendation. J Cancer Prev.

